# Hay Fever and Its Cure

**Published:** 1885-10

**Authors:** 


### Hay Fever and its Cure.

Hay fever people will be glad to see the nature and treatment of their complaint described in simple terms, readily understood by every one who comprehends the author's meaning :

These unhappy individuals, according to Dr. Sajous, of Philadelphia, possess, "as a result of hereditary or of disease implicating markedly the nervous system, nerve centers which have become abnormally sensitive, and are therefore inordinately influenced by the external elements to which they respond."

As a result of local disease the nasal mucous membrane becomes hyperæsthetic, and transmits to the abnormally sensitive nerve centers the impressions made by the "external irritants" (pollen, etc.) which results in a paroxysm of "hay fever."

These are the three conditions necessary for a paroxysm, and when one is absent, as is the case with the external visitants a portion of the year, and all the year in certain regions, it will not take place. Hence to cure the disease is to render the hyperæsthetic nasal membrane oblivious to the annual visitation of the external cause. The writer maintains that this can be done by cauterizing the hyperæsthetic portions of the nasal membrane, which he has accomplished with pleasing and permanent results by means of the galvano-cautery or acids.

He describes these hyperæsthetic areas as consisting of three—posterior, middle and anterior. The posterior area is implicated when reflex asthma is the most prominent symptom ; the anterior when the head symptoms alone are present ; the middle is the starting point of all the symptoms combined.

He recommends that abnormal conditions of the nasal cavities, such as hypertrophies, polypi, exostoses, etc., be eradicated before using the special cauterization. The best results are obtained by instituting treatment six weeks at least before the onset of a paroxysm, though it may be conducted during a paroxysm, resulting sometimes in an arrest of it or a beneficial modification. Immunity depends on the thoroughness with which the treatment is conducted.

It would seem, says the *Medical Record*, from the perusal of Dr. Sajou's monograph on the subject, that hay fever might become unknown, provided its victims would put their hyperæsthetic membranes under the treatment of an adept in rhinology. The banishment of hay fever from the list of diseases would be a boon to all except the hotel keepers of those resorts where, since the "external irritant" does not lurk in the atmosphere, the cause is removed and a cure effected.

With the mechanism of the disease in mind there remains one other method, which will be as much superior to that advanced by Dr. Sajous as his is better than the now prevailing method of changing abode, namely, that of finding a remedy which will act directly upon the abnormally sensitive nerve centers. We commend this to investigators.

